# Development of an Indirect Competitive ELISA Based on a Stable Epitope of β-Lactoglobulin for Its Detection in Hydrolyzed Formula Milk Powder

**DOI:** 10.3390/foods13213477

**Published:** 2024-10-30

**Authors:** Qinggang Xie, Yuhao Huang, Xianli Zhang, Xiaoxi Xu, Zhenxing Li

**Affiliations:** 1 College of Food Science, Northeast Agricultural University, No. 600, Changjiang Road, Harbin 150030, China; xieqinggang@feihe.com; 2 Heilongjiang Feihe Dairy Co., Ltd., No. 10, Jiudianqiao Road, Beijing 100015, China; 3 College of Food Science and Engineering, Ocean University of China, No. 1299, Sansha Road, Qingdao 266003, Chinalizhenxing@ouc.edu.cn (Z.L.)

**Keywords:** β-lactoglobulin, stable epitope, ELISA, thermal process, hydrolyzed formula milk powder

## Abstract

The target of traditional immunological detection methods for milk allergens is usually the whole β-lactoglobulin molecule. However, thermal processes and hydrolysis can destroy the epitope of β-lactoglobulin and interfere with its accurate detection and labeling in prepackaged foods, posing a health risk to milk-allergic patients. There currently remains a need to excavate and locate recognition sites for β-lactoglobulin in thermally processed and hydrolyzed products. Therefore, a stable epitope of β-lactoglobulin (CAQKKIIAEKTKIPAVFKIDA) was selected as the ideal recognition site, and an indirect competitive enzyme-linked immunosorbent assay (ELISA) was developed using an antibody against this stable β-lactoglobulin epitope in order to improve the detection of β-lactoglobulin in thermally processed and hydrolyzed foods in this study. The stable epitope of β-lactoglobulin was selected using a molecular dynamics simulation, and the binding ability of anti-stable epitope antibodies was characterized using indirect ELISA and indirect competitive ELISA. The limit of detection (LOD) and limit of quantitation (LOQ) of the established ELISA were 0.25 and 1.07 mg·kg^−1^, respectively. Furthermore, the developed ELISA only showed cross-reactivity to goat milk among 23 common foods, therefore exhibiting high specificity to bovine β-lactoglobulin. In addition, the developed ELISA was able to effectively detect β-lactoglobulin residue in processed commercial foods and hydrolyzed formula milk powder. Our findings provide a novel strategy for accurately detecting milk allergens based on stable epitope recognition in thermally processed and hydrolyzed foods.

## 1. Introduction

Whey protein is the second major milk protein component and is obtained by removing coagulated casein curd from milk. As a complex mixture of various biologically active components, whey protein offers significant nutritional and functional benefits. These components include α-lactalbumin, β-lactoglobulin, immunoglobulins, bovine serum albumin, and a range of peptone proteases, all of which play distinct roles in the body’s metabolism and immune responses. Among these, α-lactalbumin and β-lactoglobulin are the predominant proteins, known for their high bioavailability and excellent amino acid profiles, making them essential for muscle repair and growth [1]. Due to its excellent solubility and nutrition, whey protein is widely applied in various formula foods [2]. However, milk is one of the eight major allergic foods identified by the Food and Agriculture Organization of the United Nations (FAO); milk allergies affect approximately 2% of infants and 4.5% of children worldwide [3]. Casein is not the only milk protein that can induce allergic reactions; whey protein can also cause allergic symptoms. According to previous research, β-lactoglobulin is the major allergen in whey protein, accounting for 82% of milk allergies [4]. Avoiding the intake of milk and milk products is currently the most effective way for milk allergy patients to avoid allergic responses, due to the lack of effective therapies [5]. However, owing to the usual sharing of production lines for different formula foods, accidental cross-contamination is frequent [6]. Accidental exposure to milk allergen residues in foods caused by mislabeling or contamination during food processing continuously threatens the health of milk-allergic individuals [7].

Effective detection is a prerequisite to the accurate labeling of milk allergens and assists milk-allergic patients in avoiding the intake of milk and its products [8]. Considering the content and frequency of triggering allergic reactions, β-lactoglobulin and caseins are ideal targets for milk allergens [9]; β-lactoglobulin is typically used as the protein marker for whey protein allergen detection. To date, several methods based on mass spectrometry or liquid chromatography, such as liquid chromatography–tandem mass spectrometry (LC-MS/MS) [10], reversed-phase high-performance liquid chromatography [11], and parallel reaction monitoring mass spectrometry [12], have been developed to detect milk allergen residues in food samples. Although the mass-spectrometry-based method is highly sensitive and accurate, its reliance on complex instruments and time-consuming pretreatments, such as enzymatic hydrolysis, limits its application in the rapid on-site detection of milk allergens in food [13]. Compared to methods based on the polymerase chain reaction, immunology-based methods detect the actual residue of the target protein rather than its corresponding nucleic acid, more directly indicating the concentration of the allergen’s residue in samples. Furthermore, immunology-based methods can rapidly, reliably, and sensitively detect targets in complex samples, which makes them suitable for on-site allergen detection.

Currently, various immunology-based methods, including enzyme-linked immunosorbent assay (ELISA) and immunochip and lateral flow immunoassay (LFIA), have been developed for β-lactoglobulin detection [6,14]. Rodríguez-Camejo et al. [15] established a highly sensitive sandwich ELISA for bovine β-lactoglobulin using a nanobody with a limit of quantitation of 40 pg/mL. Recently, a portable and reproducible quartz crystal microbalance immunochip was developed, which could detect the β-lactoglobulin allergen in milk products without the application of a label [16]. Several commercial ELISA kits have been applied for detecting caseins or β-lactoglobulin in various food matrices, including cookies, meat-based products, and chocolate [17,18,19]. However, processing, as well as the antigen–antibody matrix, could interfere with detection by these immunology-based methods, especially ELISA, for processed food samples. The indirect ELISA developed by Villa et al. [20] using anti-β-lactoglobulin antibodies produced in rabbits could not quantify the amount of β-lactoglobulin present in autoclaved sausages. The research of Sefat et al. [19] also demonstrated that processing has a significant effect on the recovery and variability of casein and β-lactoglobulin in a dark chocolate matrix. The destruction of the partial epitopes and antibody-binding sites of target antigens during processing could hinder binding or recognition between the allergen and corresponding antibody [21]. Therefore, seeking a processing-stable antibody-binding site or epitope and developing a corresponding antibody could be crucial to improving the detection performance of ELISA in processed food samples.

Molecular dynamics simulation technology could simulate the effect of temperature and pressure during processing on the changes in allergens [22]. The surface accessibility of the epitope region is usually high, which could be an advantage of applying epitopes as antibody-binding sites for allergen detection. Thus, analyzing the changes in the epitope region of β-lactoglobulin through a molecular dynamics simulation could be a novel strategy for finding a processing-stable antibody-binding site. An antibody-targeted processing-stable antibody-binding site could be applied to establish the use of ELISA for β-lactoglobulin detection in processed food. In addition to thermal processes, hydrolysis is another common method by which whey protein is processed to enhance its digestibility and absorbability [23]. Although the allergenicity of whey protein can decrease after hydrolysis processing [24], exposure to the residue of the β-lactoglobulin fraction still poses a certain risk of allergy for milk-allergic patients. Hence, it is necessary to detect the β-lactoglobulin residue in products supplemented with hydrolyzed whey protein. The β-lactoglobulin in hydrolyzed whey protein does not simultaneously possess two epitopes or antibody-binding sites; therefore, ELISA in competitive format rather than sandwich format could be more effective in detecting the β-lactoglobulin in food supplemented with hydrolyzed whey protein. Rallabhandi et al. [25] compared the performance of three sandwich ELISAs and two competitive ELISAs and found that the competitive ELISAs performed better in detecting gliadin and gluten after hydrolysis.

Therefore, the aim of the present study was to excavate the recognition sites of thermally processed and hydrolyzed β-lactoglobulin using molecular dynamics simulation technology as the ideal detection targets and further develop ELISA based on mouse anti-stable epitope antibodies to improve the detection of β-lactoglobulin in thermally processed and hydrolyzed foods. In addition, the performance parameters of the developed ELISA were determined according to the guidelines of the Association of Official Analytical Chemists (AOAC). The developed ELISA was also applied to β-lactoglobulin residue in commercially processed foods and hydrolyzed formula milk powder to validate its ability to accurately detect milk allergens in thermal processed and hydrolyzed food samples.

## 2. Materials and Methods

### 2.1. Materials

β-Lactoglobulin (Cat number: L3908) and ovalbumin (OVA, Cat number: A5378) were purchased from Sigma-Aldrich (St. Louis, MO, USA). The control cookie of the Food Analysis Performance Assessment Scheme was obtained from (FAPAS) Proficiency testing from Fera Science Ltd. (York, UK). The Protein A/G Beads 4FF column (Cat number: R8281), keyhole limpet hemocyanin (KLH, Cat number: K8160), Tween-20 (Cat number: T8220), and 3,3′,5,5′-tetramethylbenzidine (TMB) single-component substrate (Cat number: PR1200) were purchased from Beijing Solarbio Science and Technology Co., Ltd. (Beijing, China). Horseradish peroxidase (HRP)-conjugated goat anti-mouse IgG antibody (Cat number: C030269) was purchased from the Bai Aotong Experimental Materials Center (Luoyang, China).

### 2.2. Selection of the Stable Epitope of β-Lactoglobulin

β-Lactoglobulin epitopes were collected from previous studies and the IEDB database [26]. GROMACS 2019.5 software was employed to simulate the change in the β-lactoglobulin during processing using the CHARMM36 force field. Briefly, the 3D structure of β-lactoglobulin (1DV9) was obtained from the Uniprot database and then enclosed in a cuboid water box. After equilibrating to a constant volume (NVT) and temperature (NPT) for 100 ps, the temperature and pressure were set at 1 bar (298.15 K, 333.15 K, 353.15 K, and 373.15 K) and 1.04 bar (394.15 K). The program was run for 50 ns to simulate the effect of temperature and pressure on β-lactoglobulin’s structure. The root mean square fluctuations (RMSFs) of each amino acid were analyzed using GROMACS tools. An amino acid with an RMSF value lower than the average RMSF value of β-lactoglobulin was defined as a stable amino acid. The stable epitope of β-lactoglobulin was selected according to the proportion of stable amino acids.

### 2.3. Preparation and Characterization of the Anti-Stable Epitope Polyclonal Antibody

#### 2.3.1. Synthesis and Verification of the Stable Epitope Peptide

Three selected stable epitopes of β-lactoglobulin peptides were prepared using Fmoc solid-phase synthesis by ProteinGene Biotech Co. (Wuhan, China) and then validated using liquid chromatography–tandem mass spectrometry.

#### 2.3.2. Preparation and Purification of the Anti-Stable Epitope Polyclonal Antibody

After coupling to KLH, the KLH-stable epitope peptide was used as an immunogen to immunize 6–8-week-old Balb/c female mice on days 1, 28, 42, 47, and 54 with 50 μg each time. The Protein A/G Beads 4FF column was employed to purify and prepare the mouse anti-stable epitope polyclonal antibody. All the animal-related protocols including feeding, immunization, and sacrifice were carried out following the guidelines of and were approved by Ocean University of China’s ethics committee (OUC-AE-2023098).

#### 2.3.3. Evaluation of the Binding Ability of the Anti-Stable Epitope Polyclonal Antibody

The ability of three mouse anti-stable epitope polyclonal antibodies to bind to β-lactoglobulin was characterized by using indirect ELISA and indirect competitive ELISA. In the indirect ELISA, β-lactoglobulin (5 μg·mL^−1^) diluted in carbonate salt buffer (CBS) with a pH of 9.6 was used to coat 96-well plates at 4 °C overnight. Then, 150 μL of 0.01 M phosphate salt buffer containing 0.05% Tween-20 (PBST) and 1% OVA was added per well, and the mixture was incubated for 2 h for blocking at 37 °C. Afterward, three mouse anti-stable epitope polyclonal antibodies diluted with PBST in concentration series were used as primary antibodies and incubated with the plate (100 μL per well) for 2 h at 37 °C. The serum of mice immunized with normal saline was used as a negative control. Subsequently, an HRP-conjected goat anti-mouse IgG antibody (Solarbio, Beijing, China) was diluted 1:5000 (*v*/*v*) with PBST and incubated with the plate (100 μL per well) for 1 h at 37 °C. Finally, 100 μL of TMB single-component substrate per well was incubated with the plate for 10 min at 37 °C. After terminating the reaction using 2 M sulfate acid (50 μL per well), a BioTek 800 TS microplate reader (Agilent, Santa Clara, CA, USA) was employed to measure the optical density (OD) at 450 nm. The plate was washed using PBST three times (5 min per time) between each step. The P/N value was calculated as the OD value of the mouse anti-stable epitope polyclonal antibodies divided by the OD value of the negative control.

In the indirect competitive ELISA, three mouse anti-stable epitope polyclonal antibodies were preincubated with β-lactoglobulin diluted in concentration series at 37 °C for 20 min before using them as primary antibodies. The rest of the steps were the same as those for the indirect ELISA. The inhibition rate was calculated as follows:

(1) $$Inhibiton\;rate\left(\%\right)=\frac{{OD}_{control}-{OD}_{sample}}{{OD}_{control}}\times 100$$

where *OD_control_* represents the OD of the serum of mice immunized with normal saline at 450 nm, and *OD_sample_* represents the OD for the three mouse anti-stable epitope polyclonal antibodies diluted with PBST in concentration series at 450 nm.

### 2.4. Establishment of the Competitive ELISA Using the Anti-Stable Epitope Polyclonal Antibody

β-Lactoglobulin (5 μg·mL^−1^) in CBS (pH 9.6) was used to coat an ELISA plate overnight at 4 °C, with 100 μL per well. Then, 150 μL of PBST containing 1% OVA was added per well for blocking at 37 °C for 2 h. Afterward, the mouse anti-BLG-2 antibody (78 ng·mL^−1^) was preincubated with 0, 0.1, 1, 5, 10, 50, 100, and 500 μg·mL^−1^ β-lactoglobulin at a ratio of 4:1 (*v*/*v*) at 37 °C for 20 min. Next, a mixture of mouse anti-BLG-2 antibodies and β-lactoglobulin was added to the plate (100 μL/well), and the plate was incubated at 37 °C for 30 min. Subsequently, 100 μL of the secondary antibody (HRP-conjugated goat anti-mouse IgG antibody diluted using PBST at a ratio of 1: 5000) was added to each corresponding well, and the plate was incubated for 30 min at 37 °C. After that, 100 μL of TMB substrate was added per well and reacted in the dark for 10 min before we then added 2 M sulfate acid (50 μL per well) to stop the reaction. Finally, a BioTek 800 TS microplate reader (Agilent, Santa Clara, CA, USA) was employed to measure the OD at 450 nm. The plate was washed using PBST three times (5 min per time) between each step. The OD of β-lactoglobulin in a concentration series at 450 nm is denoted as *B*, while the OD of the control buffer (PBS, pH 7.4) at 450 nm is denoted as *B*_0_. The concentration of β-lactoglobulin was fitted against *B*/*B*_0_ to form a standard curve.

The protocol for the sample extraction was as follows. The sample was homogenized in the extraction buffer (50 mM CBS containing 0.1% Tween-20) at a ratio of 1:10 (*v*/*v*) for 20 min at 60 °C. Then, the mixture was centrifuged at 4000 rpm for 15 min at room temperature, and the supernatant was collected for further measurement.

### 2.5. Performance Evaluation of the Indirect Competitive ELISA

#### 2.5.1. Validation of Sensitivity

PBST (pH 7.4) was used as the blank control and then tested using the developed competitive ELISA in ten replicates. The mean value of the determined β-lactoglobulin concentration plus 3 and 10 times the standard deviation were defined as the LOD and LOQ of the developed competitive ELISA, respectively.

#### 2.5.2. Validation of Specificity

The crude proteins of goat milk, horse milk, donkey milk, camel milk, wheat, rice, corn, soybean, pea, red bean, hazelnut, peanut, walnut, sesame, pistachio, beef, mutton, chicken, shrimp, perch, celery, onion, and egg white were extracted as described in Section 2.4. After centrifugation and collection, the supernatant was measured using the developed ELISA to validate its specificity.

#### 2.5.3. Recovery in the Food Matrix

To determine the recovery of the developed competitive ELISA in the food matrix, β-lactoglobulin (10, 100, and 1000 μg·g^−1^) was used to spike three blank food matrices (cookies, bread, and jujube cake). After extraction as described in Section 2.4, the β-lactoglobulin residue in the food matrix was measured using the developed ELISA. Recovery was calculated as the ratio of the β-lactoglobulin level measured in the model food matrix to the corresponding spiked β-lactoglobulin level.

#### 2.5.4. Precision

The concentration of β-lactoglobulin in the control cookie of the Food Analysis Performance Assessment Scheme (FAPAS) was measured using the developed ELISA to evaluate its precision. Twenty-five extracts of one sample were determined in one test to calculate the intra-assay reproducibility of the developed ELISA. In addition, five extracts of one sample were determined on five different days to calculate the inter-assay reproducibility of the developed ELISA.

#### 2.5.5. Detection of Commercial Foods and Hydrolyzed Formula Milk Powder

Fourteen commercial foods and fourteen kinds of hydrolyzed milk powder with different hydrolysis degrees were bought from Aeon Supermarket. The commercial food and hydrolyzed milk powder were extracted as described in Section 2.4 and then measured using the developed ELISA to determine their performance in practice.

### 2.6. Statistical Analysis

The experimental data were analyzed and plotted using SPSS 25.0 and Origin 2024b. Data are presented as the mean ± standard deviation (*n* = 3) unless otherwise specified. Statistical significance was analyzed using the ANOVA method, with *p* < 0.05 considered indicative of a statistically significant difference.

## 3. Results and Discussion

### 3.1. Choice of the Stable Epitope of β-Lactoglobulin

**Figure 1:**
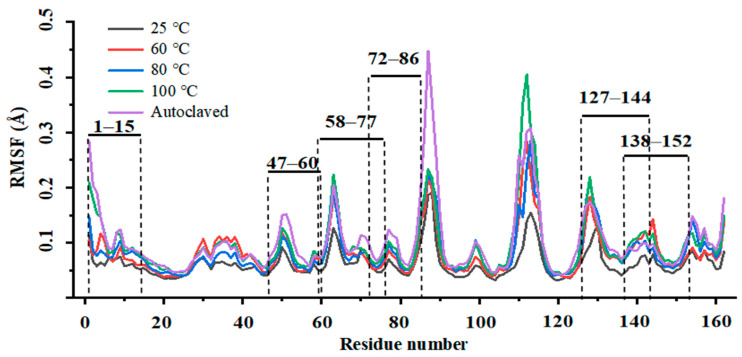
Molecular dynamics simulation of β-lactoglobulin. The black, red, blue, green, and purple lines represent simulation at 298.15 K (25 °C), 333.15 K (60 °C), 353.15 K (80 °C), and 373.15 K (100 °C) for 1 bar and 394.1 K (121 °C) for 1.04 bar.

As shown in Table 1, six epitopes (E1, E2, E3, E4, E5, and E6) were collected from previous studies and the IEDB database. The RMSF represents the degree of the displacement of the corresponding amino acid during the process simulation [27]. A lower RMSF indicates that a particular amino acid is more stable during the process. The molecular dynamics simulation for β-lactoglobulin demonstrated that the RMSFs in the E1 and E5 regions were high (Figure 1). Thus, E1 and E5 were not chosen as the stable epitope because the proportions of the stable amino acids of E1 (53%) and E5 (27%) were lower than 60%. E2 had a higher proportion of stable amino acids (71%) and was chosen as the stable epitope, named BLG-1. In addition, E3 overlapped with E4 in region AA 72–77. Therefore, E3 and E4 were fused, and the region AA 58–65, which had a higher proportion of non-stable amino acids, was removed to form a non-overlapping stable fusion epitope, named BLG-2. The proportion of stable amino acids in E6 was 66% (over 60%). Thus, E6 was chosen as the third stable epitope, named BLG-3.

Before synthesizing the corresponding epitope peptides, one cysteine was supplemented at the C-terminals of the three selected stable epitopes (BLG-1, BLG-2, and BLG-3) for further coupling to KLH using m-maleimidobenzoyl-N-hydroxysuccinimide. Liquid chromatography indicated that the purity of the synthesized epitope peptides was over 95% (Figure S1). In addition, the masses of the synthesized epitope peptides were consistent with the corresponding theoretical molecular masses according to the electrospray ionization mass spectrometry (Figure S2). These results indicate that the synthesized stable epitope peptides could be used for the following coupling and antibody preparation.

### 3.2. Evaluation and Selection of the Anti-Stable Epitope Polyclonal Antibody

**Figure 2:**
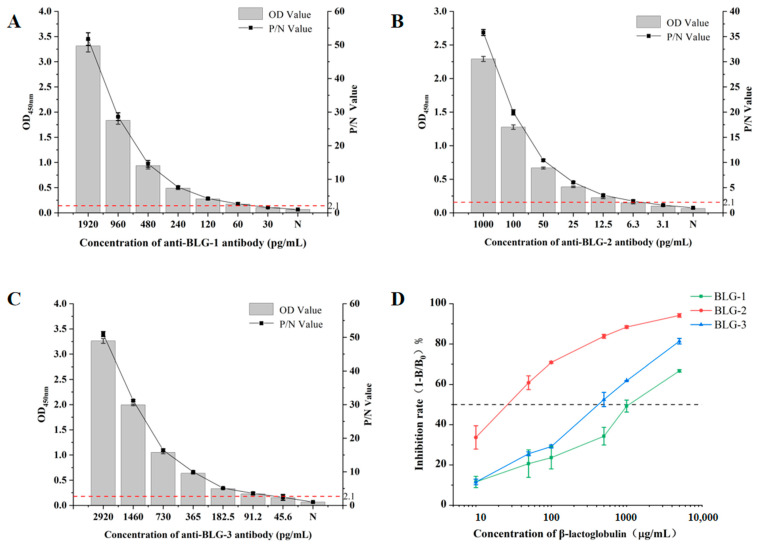
Evaluation of the binding ability of three anti-epitope antibodies using indirect enzyme-linked immunosorbent assay (ELISA) and inhibition ELISA. The binding ability of the following antibodies was validated using indirect ELISA: (**A**) anti-BLG-1; (**B**) anti-BLG-2; (**C**) anti-BLG-3. (**D**) The binding ability of the three anti-epitope antibodies was validated using inhibition ELISA.

Indirect ELISA and indirect competitive ELISA were applied to evaluate the recognition ability of three purified mouse anti-stable epitope polyclonal antibodies. In the indirect ELISA, all three mouse anti-stable epitope polyclonal antibodies could bind to β-lactoglobulin (Figure 2). The OD_450nm_ decreased with reductions in the concentrations of the three mouse anti-stable epitope polyclonal antibodies. The P/N value was applied to further quantitatively analyze the antigen-binding ability of these three antibodies. An antibody was only considered effective when its P/N value was over 2.1. As shown in Figure 2, the lowest concentrations of the mouse anti-BLG-1, BLG-2, and BLG-3 polyclonal antibodies that met this requirement were 60, 6.3, and 45.6 ng·mL^−1^, respectively. Among the three mouse anti-stable epitope polyclonal antibodies, the mouse anti-BLG-2 antibody displayed the strongest ability to bind to β-lactoglobulin. The mouse anti-BLG-1 and mouse anti-BLG-3 antibodies exhibited similar abilities to bind to β-lactoglobulin.

In the indirect competitive ELISA, the inhibition rates of the three mouse anti-stable epitope polyclonal antibodies increased with a rising concentration of β-lactoglobulin (Figure 2D). The IC_50_ of the mouse anti-BLG-2 polyclonal antibody was lower than 50 μg·mL^−1^, lower than that of the mouse anti-BLG-1 (higher than 1000 μg·mL^−1^) and mouse anti-BLG-3 (higher than 100 μg·mL^−1^) polyclonal antibodies. This demonstrated that the mouse anti-BLG-2 polyclonal antibody was more sensitive to a change in β-lactoglobulin concentration than the mouse anti-BLG-1 and anti-BLG-3 polyclonal antibodies. According to the above results, the mouse anti-BLG-2 polyclonal antibody was the best option for the establishment of the indirect competitive ELISA for β-lactoglobulin detection.

### 3.3. Validation of the Performance of the Indirect Competitive ELISA

#### 3.3.1. Sensitivity

**Figure 3:**
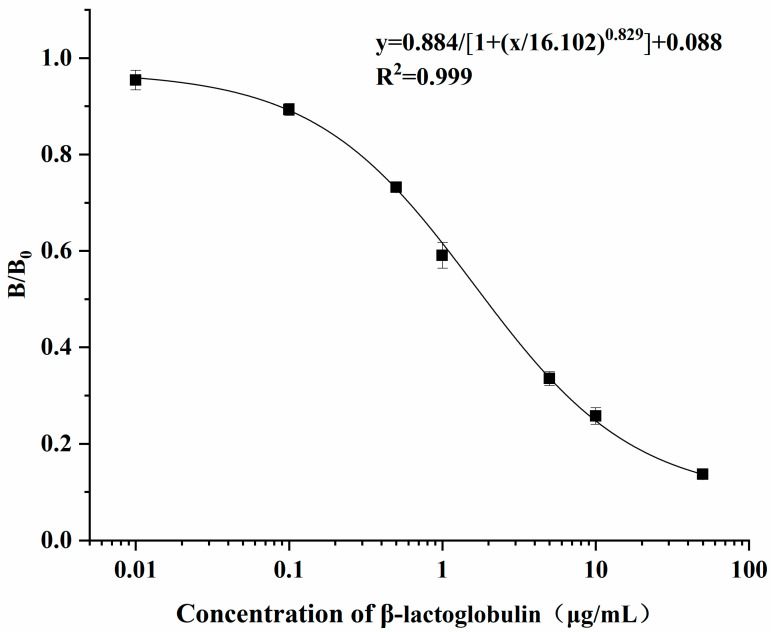
The fitted curve of the developed indirect competitive ELISA using a four-parameter logistic formula. *B* represents the measured absorbance of the serial concentrations of β-lactoglobulin at 450 nm. *B*_0_ represents the measured absorbance at 450 nm of the control dilution buffer.

The standard curve of the indirect competitive ELISA was fitted using a four-parameter logistic formula. As shown in Figure 3, the range of the curve was 0.01 to 50 μg·mL^−1^. In addition, the LOD and LOQ of the developed ELISA method were calculated to be 0.025 and 0.107 μg·mL^−1^ for β-lactoglobulin in solution, respectively. The LOD of the duplex lateral flow test developed by Galan-Malo et al. [9] was 0.5 ppm for β-lactoglobulin in the extraction buffer, which was higher than that of the developed ELISA in this study. Due to the ratio of sample to extraction buffer being 1:10 (*w*/*v*), the LOD and LOQ of the developed ELISA for β-lactoglobulin in the actual food sample were 0.25 and 1.07 mg·kg^−1^, respectively, lower than those of the competitive ELISA (0.5 mg·kg^−1^) developed by De Luis et al. [28]. Considering that the proportion of β-lactoglobulin in the milk protein was about 10%, the corresponding LOD and LOQ of the developed ELISA could be converted into 2.5 mg of milk protein per kg of food and 10.7 mg of milk protein per kg of food. According to the recommendation of the World Health Organization (WHO)/FAO proposed in risk assessment reports, the desired LOQs for cookies/biscuits and chocolate were 13.3 and 16 mg of milk protein per kg of food, respectively [29]. Thus, the sensitivity of the developed ELISA could meet the requirements for the detection of milk protein and milk allergen.

Currently, several ELISA methods have been established to detect β-lactoglobulin in food samples. De Luis et al. [28] simultaneously established sandwich and indirect competitive ELISAs for the detection of β-lactoglobulin in processed foods. The LOD of the sandwich ELISA was 0.05 mg·kg^−1^, which was 10-fold lower than that of the indirect competitive ELISA (0.5 mg·kg^−1^). R-Biopharm Company also developed two forms of commercial ELISA kits named RIDASCREEN^®^FAST β-Lactoglobulin (Art. No: R4912) and RIDASCREEN^®^ β-Lactoglobulin (Art. No: R4901) to detect β-lactoglobulin in food samples. The sandwich commercial ELISA kit also exhibits a lower LOD (mean, 0.042 mg·kg^−1^) than the competitive ELISA kit (mean, 1.4 mg·kg^−1^). These phenomena support the idea that the sandwich ELISA could be more sensitive than the competitive ELISA when detecting the whole β-lactoglobulin molecule in a sample. In principle, the sandwich ELISA requires the targeting of at least two different antibody-binding sites to form an antibody–antigen–antibody complex. However, the fragments of β-lactoglobulin in hydrolyzed milk samples do not usually possess two different antibody-binding sites, due to the destruction of the epitope during hydrolysis [30]. Thus, competitive ELISA could be more effective than sandwich ELISA for detecting the residues of hydrolyzed β-lactoglobulin fragments in a hydrolyzed milk sample. Compared to the previously developed competitive ELISA or corresponding commercial kit, the competitive ELISA developed in this study exhibits a lower LOD and excellent sensitivity.

#### 3.3.2. Specificity

The protein of nineteen non-milk foods was extracted to evaluate the specificity of the developed ELISA. As shown in Table 2, the concentrations of β-lactoglobulin determined for all nineteen non-milk foods were lower than the LOQ, which indicated that the developed ELISA exhibited no cross-reactivity to their proteins. In addition, cross-reactivity to mammalian milk such as goat, buffalo, horse, and donkey milk has been reported by previous studies for commercial kits [31], due to the high sequence similarity between the β-lactoglobulins from these mammals. Thus, the cross-reactivity to goat, horse, donkey, and camel milk was evaluated in this study. The results showed that only goat milk could be detected, which indicated that the ELISA developed in this study was more specific than the sandwich ELISA developed by Khan et al. [32] and the commercial kits. The antibody used in the developed ELISA was targeted to a single epitope (BLG-2) of β-lactoglobulin from cow’s milk. The differences in amino acids or burying of the BLG-2 epitope regions in the β-lactoglobulins of horses, donkeys, and camels could have been responsible for the good specificity of the developed ELISA.

#### 3.3.3. Recovery and Precision

The food matrix can hinder the extraction of a detection target from a sample and interfere with an antibody’s recognition of its antigen, leading to false-negative or positive detection results [33]. Previous research reported by Khuda et al. [18] demonstrated that the recovery levels of β-lactoglobulin in cookie dough were 15%, 437%, and 9% when using commercial kits developed by R-Biopharm, Morinaga, and Tepnel, respectively. Therefore, three model foods (cookies, bread, and jujube cake) that did not contain β-lactoglobulin were selected for spiking experiments to evaluate the influence of the food matrix on the established competitive ELISA. As shown in Table 3, the β-lactoglobulin recovery rates in biscuits were 84.41% to 115.24% with our method, indicating that it was less susceptible to matrix interference than the previously reported commercial kits. In addition, the β-lactoglobulin recovery rates in bread and date cakes were 95.67% to 118.33% and 95.03% to 114.21%, with a coefficient of variation lower than 20%, which were acceptable ranges according to the recommended recovery rate (80–120%) of the guidelines of the AOAC [34].

In order to validate the precision of the developed ELISA, the intra- and inter-assay reproducibility were determined using the control cookie of FAPAS. As shown in Table 4, the intra-assay coefficient of variation ranged from 5.56% to 8.44%, remaining less than 10%. The inter-assay coefficient of variation ranged from 5.74% to 13.59%, remaining less than 20%. All the precision parameters were within the performance requirements of ELISA for allergen detection, according to the AOAC guidelines [34]. Therefore, the developed ELISA could be considered to comply with the principle of standardization. The ELISA developed in this study was more precise than the previously reported sandwich ELISA, which had intra- and inter-assay coefficients of variation ranging from 4.02% to 14.62% and 6.05% to 15.08%, respectively.

#### 3.3.4. Detection of Milk Protein Residues in Commercial Foods and Hydrolyzed Formula Milk Powder

In order to evaluate the performance of the developed competitive ELISA in practice, fourteen commercial foods were employed for extraction and detection. According to the allergen label descriptions on the food packaging, these fourteen commercial foods could be divided into three categories: labeled as containing milk or whey (six), labeled as possibly containing milk or whey (four), and labeled as not containing milk or whey (four). As shown in Table 5, β-lactoglobulin could be detected in all six commercial foods labeled as containing milk or whey, at concentrations ranging from 2.13 to 955.23 mg·kg^−1^. This indicated that the developed competitive ELISA exhibited excellent performance in detecting β-lactoglobulin in thermally processed foods. Regarding commercial foods labeled as possibly containing milk or whey, β-lactoglobulin could be detected in black chocolate. The abundant lipids and polyphenols in the black chocolate matrix could covalently bind to the target protein, which could interfere with the extraction of the target protein and binding between the antibody and target protein [19]. However, the developed competitive ELISA could effectively detect the β-lactoglobulin residue in black chocolate. This could be because the BLG-2 epitope was not buried by lipids and polyphenols. In addition, the concentrations of β-lactoglobulin determined in all four commercial foods labeled as not containing milk or whey were lower than the LOQ, demonstrating good specificity for the developed competitive ELISA. These results support the idea that the β-lactoglobulin residue in commercial foods can be accurately detected using the developed ELISA. 

The established competitive ELISA using the anti-stable epitope antibody was also used to screen hydrolyzed formula milk powder. As shown in Table 6, the developed competitive ELISA could detect the residue of the whey protein allergen β-lactoglobulin whether the formula milk products were partially or deeply hydrolyzed. Among the five partially hydrolyzed formula milk powder products, the determined β-lactoglobulin concentration varied widely from 11.60 to 11,047.37 mg·kg^−1^. This may have been due to the different methods by which different partially hydrolyzed formula milk powder products are hydrolyzed, resulting in varying degrees of the deconstruction of the BLG-2 epitope in β-lactoglobulin. The determined concentrations of β-lactoglobulin in the deeply hydrolyzed formula milk powder products ranged from 0.54 to 7.56 mg·kg^−1^, lower than those of partially hydrolyzed formula milk powder products. In addition, the detection results for three of Arla’s hydrolyzed formula milk powder products labeled with specific hydrolysis degrees also supported the idea that the determined concentration of β-lactoglobulin residue decreased with an increase in hydrolysis degree. These results demonstrate that the competitive ELISA could effectively detect the β-lactoglobulin residue in hydrolyzed formula milk powder, even if the hydrolysis degree of the product reached 25–30%.

Rallabhandi et al. [25] also reported that competition ELISA is more suitable for detecting hydrolyzed proteins (gliadin and glutenin) because the epitopes of allergen proteins could be destroyed during the hydrolysis process. In addition, large-molecular-weight proteins could be degraded into small-molecular-weight peptides. It is hard for small-molecular-weight peptides to bind to two antibodies simultaneously due to steric hindrance, which further results in the better recognition of allergic residues in allergen hydrolysates with the competition ELISA. Therefore, the detection results of the sandwich ELISA method and the competition ELISA could be significantly different [35,36]. In summary, the developed competitive ELISA based on anti-stable epitope antibodies could accurately and effectively detect the residue of the major whey allergen β-lactoglobulin in whey protein foods processed under harsh conditions, especially partially and deeply hydrolyzed whey protein formula products.

## 4. Conclusions

In this study, the stable recognition sites (epitope) of thermally processed and hydrolyzed β-lactoglobulin were excavated and applied as ideal detection targets to further develop ELISA based on mouse anti-stable epitope antibodies for the detection of β-lactoglobulin in thermally processed and hydrolyzed foods. The LOD and LOQ of the established ELISA were 0.25 and 1.07 mg·kg^−1^ in a food sample, respectively. The developed ELISA exhibited excellent specificity, with no cross-reactivity to nineteen non-milk foods. In addition, the recovery in three food matrices and intra- and inter-assay coefficients of variation of the developed ELISA were 84.41% to 118.33%, 5.56% to 8.44%, and 5.74% to 13.59%, respectively. The residue of β-lactoglobulin or its fraction was able to be effectively detected in all six processed commercial foods labeled as containing milk or whey and all twelve hydrolyzed formula milk powders using the developed ELISA. Our research proposes a novel strategy for the excavation of a stable epitope of β-lactoglobulin and developed a practical tool for milk allergen detection in thermally processed and hydrolyzed foods.

## Figures and Tables

**Table 1:** Selection of stable epitope of β-lactoglobulin.

Name	Epitope Sequence	Position	Stable Amino Acid Proportion	Decision
E1	LIVTQTMKGLDIQKV	1–15	8/15 (53%)	Rejected: proportion of stable amino acids lower than 60%
E2	KPTPEGDLEILLQK	47–60	10/14 (71%)	Selected as ‘BLG-1’ ^a^
E3	LQKWENDECAQKKIIAEKTK	58–77	13/20 (65%)	Fused with E4 to form ‘BLG-2’ ^a^
E4	IAEKTKIPAVFKIDA	72–86	9/15 (60%)	Fused with E3 to form ‘BLG-2’ ^a^
E5	EVDDEALEKFDKALKALP	127–144	5/18 (27%)	Rejected: proportion of stable amino acids lower than 60%
E6	KALKALPMHIRLSFN	138–152	9/15 (66%)	Selected as ‘BLG-3’ ^a^

^a^ The sequences of the three selected stable epitopes of β-lactoglobulin according to the molecular dynamics simulation were ‘KPTPEGDLEILLQK’, ‘CAQKKIIAEKTKIPAVFKIDA’, and ‘KALKALPMHIRLSFN’.

**Table 2:** Specificity assessment of the developed ELISA using nineteen non-milk proteins and four mammalian whey proteins (*n* = 3).

Protein	Concentration of β-Lactoglobulin (mg·kg^−1^)
Goat milk	5.53 ± 0.26
Horse milk	<LOQ ^a^
Donkey milk	<LOQ
Camel milk	<LOQ
Wheat	<LOQ
Rice	<LOQ
Corn	<LOQ
Soybean	<LOQ
Pea	<LOQ
Red bean	<LOQ
Hazelnut	<LOQ
Peanut	<LOQ
Walnut	<LOQ
Sesame	<LOQ
Pistachio	<LOQ
Beef	<LOQ
Mutton	<LOQ
Chicken	<LOQ
Shrimp	<LOQ
Perch	<LOQ
Celery	<LOQ
Onion	<LOQ
Egg white	<LOQ

^a^ LOQ, limit of quantitation.

**Table 3:** Recovery of β-lactoglobulin spiked in three food matrices.

Spiked Level (μg·g^−1^)	Cookie		Bread		Jujube Cake	
	Mean Recovery (%)	CV ^a^ (%)	Mean Recovery (%)	CV (%)	Mean Recovery (%)	CV (%)
1.5	84.41	10.11	95.67	7.83	114.21	4.28
15	98.69	1.66	118.33	4.79	106.80	3.52
150	115.24	2.89	115.90	4.53	95.03	6.96

^a^ CV, coefficient of variation.

**Table 4:** Precision assessment of the developed ELISA.

	2 μg·g^−1^		20 μg·g^−1^		200 μg·g^−1^	
	Mean (μg·g^−1^)	CV ^a^ (%)	Mean (μg·g^−1^)	CV (%)	Mean (μg·g^−1^)	CV (%)
Reproducibility intra-assay	1.96	8.44	20.46	5.56	197.11	7.88
Reproducibility inter-assay	2.14	13.59	19.33	5.74	191.27	9.61
Day 1	2.13		20.52		194.36	
Day 2	2.23		19.77		195.28	
Day 3	2.16		19.42		193.44	
Day 4	2.11		19.12		190.56	
Day 5	2.01		19.40		191.77	

^a^ CV, coefficient of variation.

**Table 5:** Commercial food detection using the developed ELISA. The concentration of β-lactoglobulin in the sample is expressed as mg of β-lactoglobulin per kg of food (*n* = 3).

Label	Commercial Food	β-Lactoglobulin (mg·kg^−1^)
Contained milk or whey	Waffle	2.13 ± 0.70
	Cookie	4.30 ± 0.44
	Honey-flavored potato chips	234.14 ± 27.53
	Jelly	83.62 ± 4.61
	Soda crackers	2.71 ± 0.36
	Milk cereal	955.23 ± 89.13
Might contain milk or whey	Shrimp-flavored biscuit	<LOD
	Scallop	<LOD
	Chicken essence	<LOD
	Black chocolate	83.22 ± 6.45
Did not contain milk or whey	Sesame paste	<LOD
	Ketchup	<LOD
	Noodles	<LOD
	Rice cake	<LOD

**Table 6:** Detection of β-lactoglobulin in commercially available hydrolyzed milk powder formulas. The concentration of β-lactoglobulin in the sample is expressed as mg of β-lactoglobulin per kg of hydrolyzed formula milk powder (*n* = 3).

Hydrolyzed Formula Milk Powder	Main Competent	Hydrolysis Degree	β-Lactoglobulin (mg·kg^−1^)
iSainte	Whey protein	Partial hydrolysis	11,047.37 ± 1073.78
Similac	Whey protein	Partial hydrolysis	135.96 ± 7.83
Enfamil	Milk protein	Partial hydrolysis	340.20 ± 16.73
Beingmate	Whey protein	Partial hydrolysis	947.37 ± 200.05
Nestle NAN	Whey protein	Partial hydrolysis	11.60 ± 0.61
YeePer	Whey protein	Deep hydrolysis	7.56 ± 0.71
Nestle ALFARE	Whey protein	Deep hydrolysis	2.89 ± 0.42
Nestle ALTHERA	Whey protein	Deep hydrolysis	2.13 ± 0.34
Nutramigen LGG	Milk protein	Deep hydrolysis	0.54 ± 0.03
Arla	Whey protein	9% to 15%	87,882.93 ± 5306.97
Arla	Whey protein	17% to 25%	28.83 ± 1.79
Arla	Whey protein	25% to 30%	22.97 ± 1.26

## Data Availability

The original contributions presented in the study are included in the article/Supplementary Materials; further inquiries can be directed to the corresponding authors.

## Supplementary Materials

The following supporting information can be downloaded at https://www.mdpi.com/article/10.3390/foods13213477/s1. Figure S1: Liquid chromatogram of synthetic epitope peptides. A, B, and C are the liquid chromatograms of the BLG-1, BLG-2, and BLG-3 epitope peptides. Figure S2: Synthetic epitope peptide mass spectrometry identification map. A, B, and C are the mass spectra of the BLG-1, BLG-2, and BLG-3 epitope peptides. Figure S3: Diagram of the stable epitope peptide synthesis, peptide coupling, antibody production, ELISA construction, and validation.
