# Supplementary material for: Development of an Indirect Competitive ELISA Based on a Stable Epitope of β-Lactoglobulin for Its Detection in Hydrolyzed Formula Milk Powder
Source: Foods. 2024 Oct 30;13(21):3477. doi: 10.3390/foods13213477 (PMC11545030; doi:10.3390/foods13213477)
Supplement: Supplementary file 1 [file foods-13-03477-s001.zip › foods-3234528-SI.pdf]

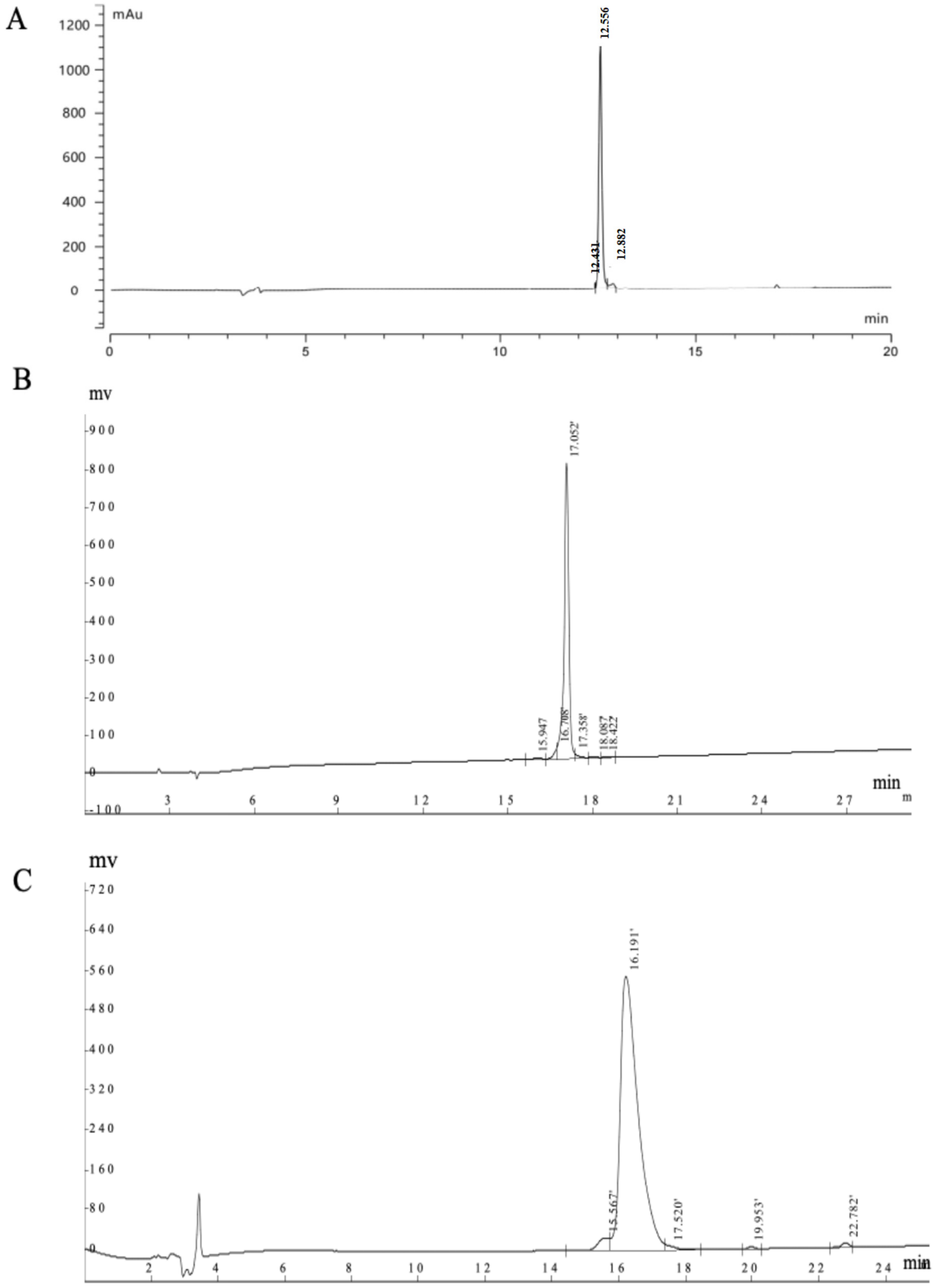

Figure S1. Liquid chromatogram of synthetic epitope peptides. A, B and C were the liquid chromatograms of BLG-1, BLG-2 and BLG-3 epitope peptides.

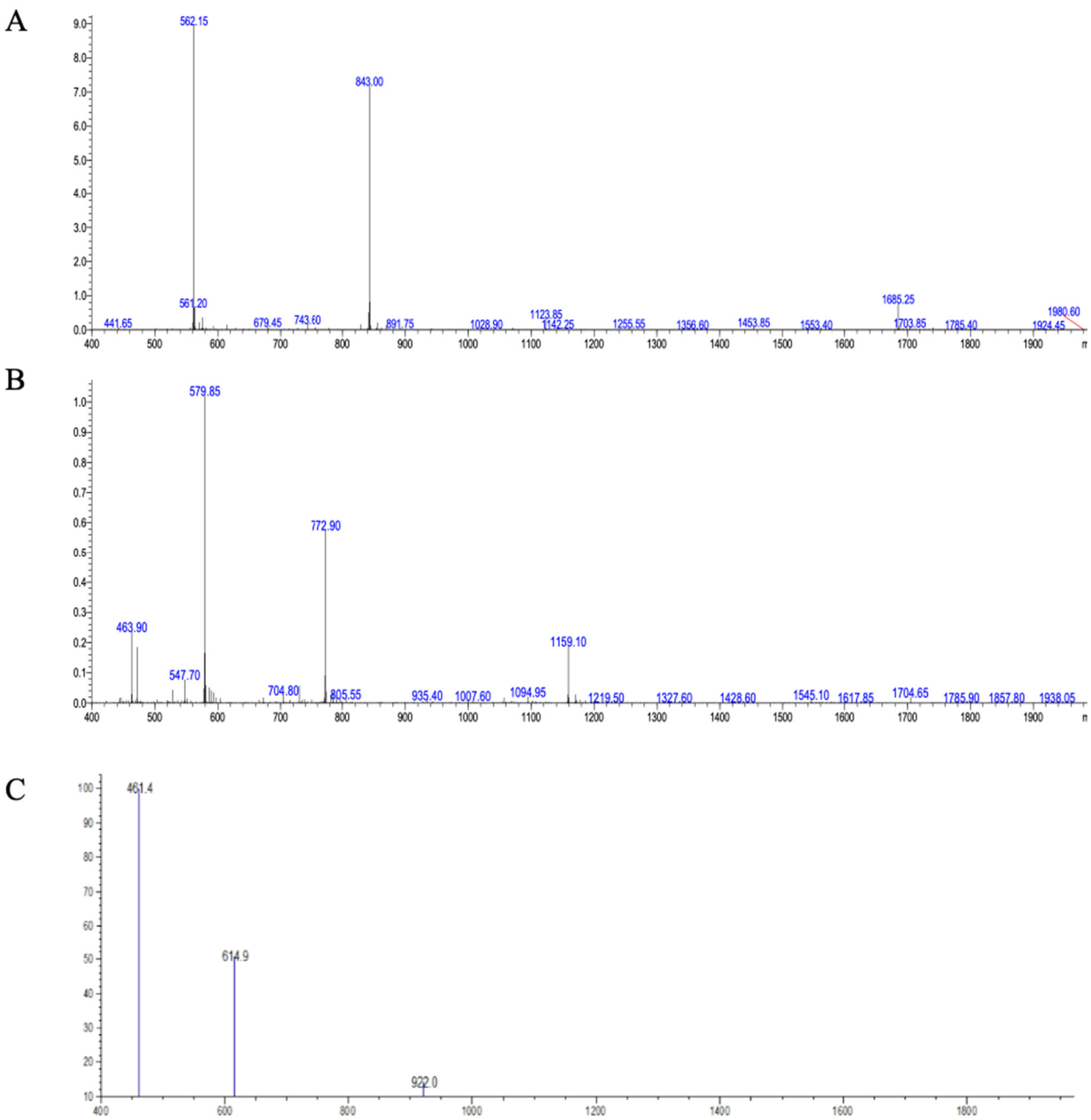

Figure S2. Synthetic epitope peptide mass spectrometry identification map. A, B and C were mass spectra of BLG-1, BLG-2 and BLG-3 epitope peptides.

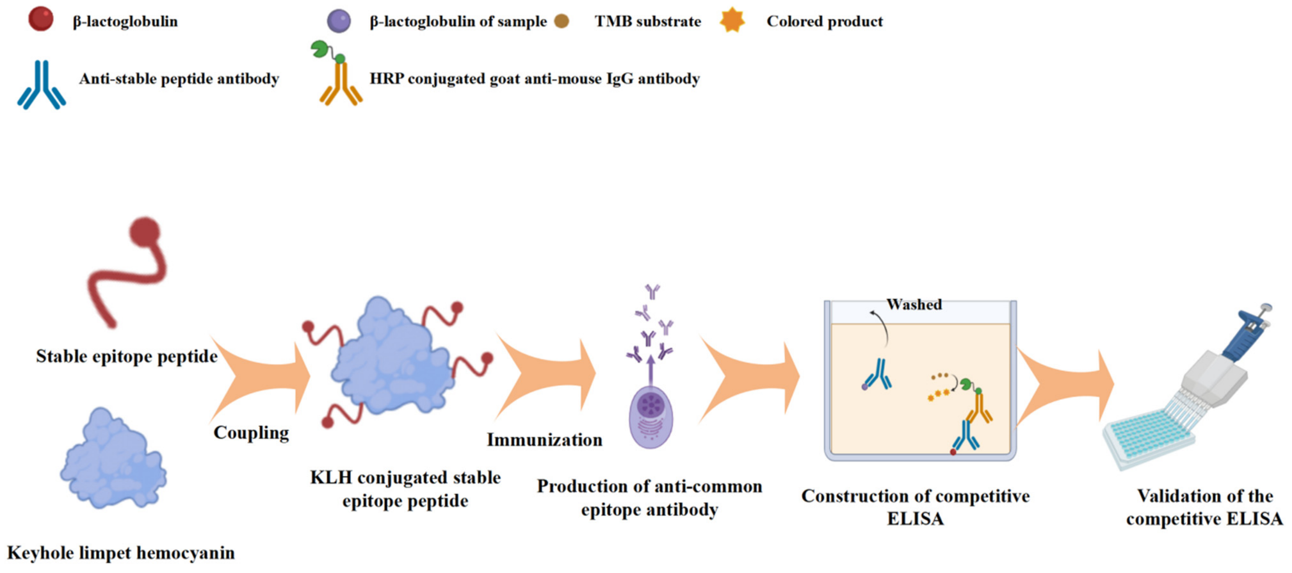

Figure S3. Diagram of the process of stable epitope peptide synthesis, peptide coupling, antibody produce, ELISA construction and validation.
